# Delay Discounting in Established and Proposed Behavioral Addictions: A Systematic Review and Meta-Analysis

**DOI:** 10.3389/fnbeh.2021.786358

**Published:** 2021-11-26

**Authors:** Sarah Weinsztok, Sarah Brassard, Iris Balodis, Laura E. Martin, Michael Amlung

**Affiliations:** ^1^Cofrin Logan Center for Addiction Research and Treatment, University of Kansas, Lawrence, KS, United States; ^2^Peter Boris Centre for Addictions Research, McMaster University, Hamilton, ON, United States; ^3^Department of Population Health, University of Kansas Medical Center, Kansas City, KS, United States; ^4^Hoglund Biomedical Imaging Center, University of Kansas Medical Center, Kansas City, KS, United States; ^5^Department of Applied Behavioral Science, University of Kansas, Lawrence, KS, United States

**Keywords:** delay discounting, behavioral addiction, systematic review, meta-analysis, behavioral economics

## Abstract

Steep delay discounting, or a greater preference for smaller-immediate rewards over larger-delayed rewards, is a common phenomenon across a range of substance use and psychiatric disorders. Non-substance behavioral addictions (e.g., gambling disorder, internet gaming disorder, food addiction) are of increasing interest in delay discounting research. Individual studies have reported steeper discounting in people exhibiting various behavioral addictions compared to controls or significant correlations between discounting and behavioral addiction scales; however, not all studies have found significant effects. To synthesize the published research in this area and identify priorities for future research, we conducted a pre-registered systematic review and meta-analysis (following PRISMA guidelines) of delay discounting studies across a range of behavioral addiction categories. The final sample included 78 studies, yielding 87 effect sizes for the meta-analysis. For studies with categorical designs, we found statistically significant, medium-to-large effect sizes for gambling disorder (Cohen’s *d* = 0.82) and IGD (*d* = 0.89), although the IGD effect size was disproportionately influenced by a single study (adjusted *d* = *0.53* after removal). Categorical internet/smartphone studies were non-significant (*d* = 0.16, *p* = 0.06). Aggregate correlations in dimensional studies were statistically significant, but generally small magnitude for gambling (*r* = 0.22), internet/smartphone (*r* = 0.13) and food addiction (*r* = 0.12). Heterogeneity statistics suggested substantial variability across studies, and publication bias indices indicated moderate impact of unpublished or small sample studies. These findings generally suggest that some behavioral addictions are associated with steeper discounting, with the most robust evidence for gambling disorder. Importantly, this review also highlighted several categories with notably smaller effect sizes or categories with too few studies to be included (e.g., compulsive buying, exercise addiction). Further research on delay discounting in behavioral addictions is warranted, particularly for categories with relatively few studies.

## Introduction

Delay discounting refers to the tendency to devalue rewards as a function of the delay to their receipt (Rachlin and Green, 1972; Madden and Bickel, 2010; Odum, 2011). In behavioral economics, delay discounting is an index used to conceptualize the overvaluation of smaller, immediate rewards over larger, delayed rewards (Bickel et al., 2014), Delay discounting is generally assessed by providing an individual with a series of choices between a small amount of a commodity (e.g., money, cigarettes, food) which is available immediately vs. a larger amount of the given commodity only obtainable after a certain delay (e.g., “would you prefer $40 today or $200 in 6 months?”). Researchers systematically vary either the commodity amount or the magnitude of the immediate and delayed rewards (e.g., $40 today or $200 in 6 months, $75 today or $200 in 6 months). Researchers will also vary the length of the delay (e.g., 1 month, 6 months, 1 year). Varying reward amount and delay to the larger reward across trials produces an indifference point, i.e., the amount at which the delayed reward has equivalent subjective value to the immediate reward. Plotting these indifference points across different delays generates a discounting curve with the steepness of this curve reflecting the degree of discounting. Delay discounting has been considered a measure of impulsivity in the past; however, recently researchers have begun to debate whether this is appropriate (see Strickland and Johnson, 2021, for a more thorough analysis of this issue). While resolving this debate is outside the scope of the current review, we will avoid use of the term impulsive to describe steep discounting.

Delay discounting tasks (DDTs) may be administered via a survey with a pre-established number of questions in which the reward values and delay length varies across questions like the monetary choice questionnaire (MCQ; Kirby et al., 1999). They may also be adjusting intertemporal choice tasks administered on a computer or mobile device in which the delay lengths or the reward value automatically adjusts up or down (titrates) based on the participant’s response to the previous choice. Still others provide a single choice between a smaller, immediate reward and a larger, delayed reward (i.e., “single-shot” discounting tasks). The magnitude of the immediate and delayed rewards, the length of the delays, the number of choices offered, and the commodity of interest all may differ across tasks; thus, sizeable heterogeneity exists across published delay discounting data sets.

To assess individual and group differences in delay discounting, theoretical [*k*, log(*k*), effective delay 50] or atheoretical (area under the curve, impulsive choice ratio) measures may be derived from the data. The *k* parameter is derived from exponential, hyperbolic, or hyperboloid discounting functions and quantifies the degree of discounting observed. Effective delay 50 (ED50) is the inverse measure of *k* (Yoon and Higgins, 2008) and reflects the delay at which the subjective value of the delayed reward loses 50% of its value. Traditionally, most delay discounting curves are best fit by hyperbolic or hyperboloid functions that can account for preference reversals, in other words, the phenomenon observed in which an initial preference for the smaller, immediate reward shifts to the larger, delayed reward as the delays to both the immediate and delayed reward are increased (Green et al., 1994; McKerchar et al., 2009; Odum, 2011). Quantifying the area under the curve or the proportion of choices made for the immediate reward (impulsive choice ratio) are alternative, atheoretical methods of assessing discounting (Myerson et al., 2001; Mitchell et al., 2005).

Despite differences in calculating delay discounting and deriving discounting parameters, discounting rates appear to be elevated across a wide range of addictive disorders. Because of the consistency of excessive delay discounting observed across a variety of disorders and unhealthy behaviors, delay discounting has been proposed as a trans-disease or transdiagnostic process (e.g., Bickel et al., 2012; Amlung et al., 2019). Several reviews and meta-analyses have synthesized this body of literature, primarily focusing on substance use disorders (e.g., MacKillop et al., 2011; Amlung et al., 2017) and other psychiatric and neurodevelopmental disorders (e.g., Jackson and MacKillop, 2016; Amlung et al., 2019; Lempert et al., 2019). While there is still ongoing debate as to whether measures of delay discounting can be considered a transdiagnostic process (see Bailey et al., 2021, for a recent critique), reviewing the growing body of literature on delay discounting and non-substance behavioral addictions can contribute to this discussion.

The “Substance-Related and Addictive Disorders” category in the fifth edition of the Diagnostic and Statistical Manual of Mental Disorders (*DSM-5;* American Psychiatric Association, 2013) DSM-5, introduced “behavioral addictions,” with gambling disorder recognized as the first “non-substance-related” disorder (American Psychiatric Association, 2013). In addition to gambling, many different behavior patterns have been proposed as behavioral addictions [for a comprehensive account of criteria, see Rosenberg and Feder, 2014, including, videogaming, smartphone and internet use, food consumption, sex, and compulsive buying (Holden, 2001)]. While initially pleasurable, increasing priority of these behaviors over others can lead to dysregulation, as an individual experiences negative consequences and impaired control. The DSM-5 substance-related disorders work group examined Internet gaming and other non-substance-related behaviors (e.g., shopping) other than gambling. While they found a large literature base for internet gaming, the work group concluded that additional research was still needed, and that research on other behaviors was even more preliminary (Hasin et al., 2013). Other APA working groups for addictions examined sex and eating, finding insufficient peer-reviewed evidence to classify these behaviors as addictive disorders (American Psychiatric Association, 2013). However, the state of the research demonstrated similar phenomenological and neurobiological substrates between gambling and substance use disorders, warranting the inclusion of the new classification (Frascella et al., 2010). Currently, gambling disorder is the only behavioral condition included in this category in the DSM-5, although internet gaming disorder (IGD) is now included in the ICD-11 (World Health Organization, 2018) and listed in Section III of the DSM-5 as a condition requiring additional study.

There is increasing concern that symptom-based models of addictive disorders can lead to a pathologizing of common behaviors, thereby reducing the relevance and credibility of the diagnosis (Kardefelt-Winther et al., 2017). Some have argued that the lack of a theoretical framework for behavioral addictions, such as those which exist for substance-related addictions, is cause for concern and that research on behavioral addictions should be guided by process-based as opposed to criteria-based approaches (Billieux et al., 2015). However, phenomenological, clinical, and neurobiological similarities do exist between gambling disorder and proposed behavioral addictions. For example, many risky behaviors such as gambling, hypersexuality, compulsive shopping and excessive eating have been linked to Parkinson’s disease and are related to dopamine receptor functioning, thereby suggesting a common biological pathway (e.g., Evans et al., 2009). Additionally, the clinical presentation is often that of these conditions co-occurring and individuals often seek help for these behaviors at clinics, despite no specific diagnosis or treatment for them. For these reasons, we believe that an improved understanding of these conditions is warranted.

While excessive use or engagement in a particular activity may not be enough to categorize that behavior as pathological (Billieux et al., 2015), examining these behaviors through a behavioral economic lens may provide more insight into underlying processes that warrant further investigation. A systematic review of both established and proposed behavioral addictions research is an important step toward compiling existing evidence across these behaviors to better understand the phenomena. We make these caveats because most categories of behavioral addiction present in the current review are not listed in the DSM-5; however, whether these disorders should be considered diagnosable behavioral addictions is beyond the scope of the review.

Delay discounting rates in gambling disorder and IGD have been the focus of separate meta-analyses (MacKillop et al., 2011; Amlung et al., 2017; Cheng et al., 2021; Yao et al., 2021); however, no review has synthesized findings across all proposed behavioral addictions. Therefore, the purpose of the current study was to conduct a systematic review and meta-analysis of published studies comparing delay discounting rates between individuals with non-substance behavioral addictions and healthy controls or studies assessing dimensional associations between delay discounting and quantity/frequency or severity of the behavioral addiction presented. Secondary purposes included updating and synthesizing the novel research on gambling disorder conducted since previous meta-analyses and comparing rates of delay discounting across behavioral addictions. A final purpose, based on the results of the review and meta-analysis, is identifying areas that warrant further study.

## Methods

### Literature Search and Study Selection

**FIGURE 1:**
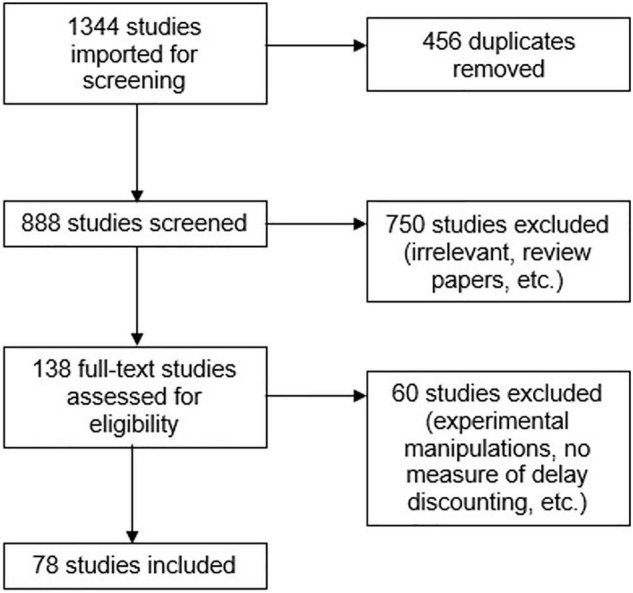
PRISMA diagram.

The current systematic review and meta-analysis was pre-registered with PROSPERO (#CRD42021257164) and followed the Preferred Reporting Items for Systematic Review and Meta-Analysis (PRISMA; Page et al., 2021) standards. Searches of PubMed and PsycInfo databases were conducted to identify studies using an all-text search strategy with keywords listed in Supplementary Table 1. Database searches were conducted through June 25, 2021 and were not restricted by year or journal (except for English language). The returned records were uploaded to Covidence^1^ (Level 10, Melbourne, Australia), an online software used to help streamline the systematic review process. To be included, studies had to meet the following criteria: (i) published in an English language peer-reviewed journal, (ii) assessed one or more types of behavioral addiction among human participants, (iii) included at least one measure of delay discounting, (iv) included either a comparison of a behavioral addiction group and a control group OR a correlation coefficient measuring the association between delay discounting and the behavioral addiction of interest. Because a formal diagnosis of “behavioral addiction” does not exist for every present category, studies were included if the authors measured engagement with the activity using an empirically validated psychometric scale that differentiated between non-problematic and problematic, excessive, or pathological use or engagement. The full study selection procedure is outlined in Figure 1.

The articles were screened for inclusion first by abstract, then by the full text, by two independent raters (SW and MA) with conflicts resolved by consensus rating at each stage. A total of 78 studies met inclusion criteria. The number of unique effect sizes in each behavioral addiction category were as follows: gambling = 53 (28 categorical, 25 dimensional), IGD = 15 (13 categorical, 2 dimensional), internet/smartphone = 16 (6 categorical, 10 dimensional), food addiction = 6 (1 categorical, 5 dimensional), and compulsive and pathological buying = 2 (1 categorical, 1 dimensional). Characteristics of included studies are presented in Supplementary Table 2. Three studies (Williams, 2012; Wölfling et al., 2020; Acuff et al., 2021) included more than one behavioral addiction category in their assessments; effect sizes from each category were included in the meta-analysis. Kräplin et al. (2020) examined a combined group of non-substance-based addictions but did not differentiate between specific categories of behavioral addictions; thus, it was omitted from the analysis.

### Data Extraction

Study characteristics, task parameters, addiction scales, and participant demographics were coded for each study. Means, standard deviations, and group ns were extracted for each categorical study. If means were not reported in text but a figure presenting these values was available, we used WebPlotDigitizer^2^ to estimate the mean and standard deviation from the high-resolution figure. Standard error values were converted to standard deviation prior to data entry. For dimensional studies, correlation values and sample sizes were extracted. In cases where data were not available in the published paper or Supplementary Materials, we contacted the authors to request data (4/5 contacted authors provided data). When reporting AUC and indifference points, a larger value indicates shallower discounting. The reverse is true for k, log(k), ln(k), or ICR. Therefore, to maintain consistency across studies, the direction of effect sizes from studies using area under the curve (AUC) or indifference points were reversed prior to analysis. Extracted data were checked for accuracy by two authors.

### Meta-Analytic Approach

Quantitative meta-analysis was conducted in Comprehensive Meta-Analysis Software Version 3.0 (Biostat, Englewood, NJ). Separate meta-analyses were conducted for each design type (categorical, dimensional) using a random-effects model. First, we estimated the aggregate effect size collapsed across all addiction types to examine the overall effect size for differences in discounting between groups or correlations with behavior addiction variables. Next, we examined each addiction category separately and calculated between-groups heterogeneity statistics to determine if effect sizes significantly differed across addiction type. Only categories with 4 or more effect sizes were included in this subgroup analysis; however, the findings of the remaining studies are described in narrative review. Several indices of effect size heterogeneity were calculated. Cochran’s Q reflects the sum of squared differences between individual weighted study effects and the overall mean. I^2^ captures the proportion of variation within study effect sizes explained by heterogeneity. Of note, Borenstein et al. (2009) emphasized that Q is less reliable with small sample sizes while I^2^ is not affected by sample size. Therefore, given the variability in number of studies per category, both statistics were reported to be comprehensive. A “one-study-removed” analysis quantified the impact of individual studies on the aggregate results (Tukey, 1958). Differences in effect sizes across different delay discounting measures were examined in a moderator analysis. This analysis was first conducted at the aggregate level for categorical and dimensional studies (collapsed across behavioral addiction type), and then repeated within each type individually. For the latter analysis, only categories with at least 4 effect sizes per level of the moderator were examined. Due to low statistical power for the funnel plot indices with small sample sizes, publication bias indices were only examined for categories with 10 or more effect sizes (Sterne et al., 2000). Indices included Orwin’s modified fail-safe N using a criterion of 50% reduction in aggregate effect size (Orwin, 1983) and examination of the funnel plots using the two-tailed Begg-Mazumdar test (Begg and Mazumdar, 1994) and the one-tailed Egger’s test (Egger et al., 1997). Finally, adjusted estimates of effect size were generated using the Duval and Tweedie (2000) trim and fill approach.

## Results

Complete demographic variables, task parameters, and other relevant characteristics from the included studies are provided in Supplementary Table 1. For those studies that did report race or ethnicity, most of the participants identified as White and non-Hispanic. For studies that reported gender, an average of 36.2% of participants reported as female. While most DDTs employed hypothetical outcomes, 9 studies provided real rewards to participants. Aside from Buono et al. (2017), all included studies used money as the only target commodity. The most common delay discounting measures used were *k* (or a log or natural log transformation of *k*) and AUC. Eleven studies utilized less-common measures such as impulsive choice ratio (ICR), total number of choices for the immediate reward, a discounting factor, indifference point, or some other derived proportion of choices of immediate and delayed rewards.

**FIGURE 2:**
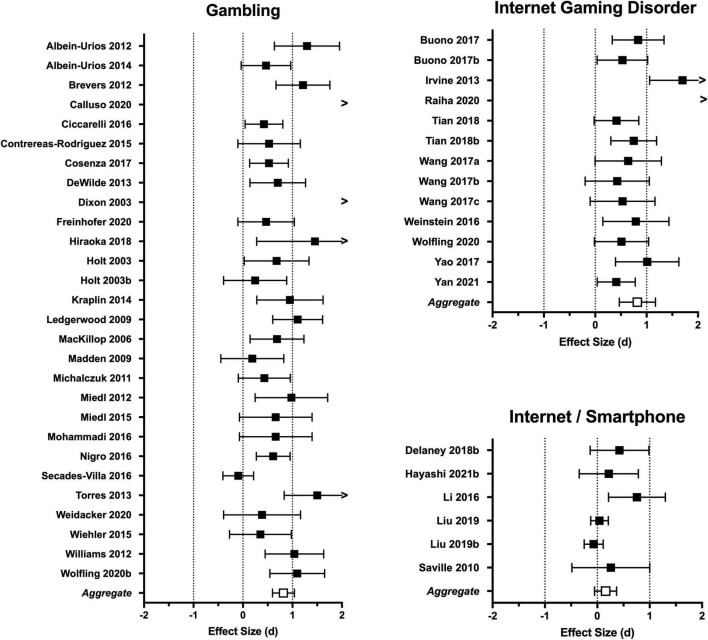
Forest plots depicting effect sizes for categorical studies. Individual data points reflect effect size (Cohen’s d) and 95% confidence intervals. The aggregate effect size generated by the random-effects model is provided at the bottom of each forest plot. Complete data is provided in Supplementary Table 3.

**FIGURE 3:**
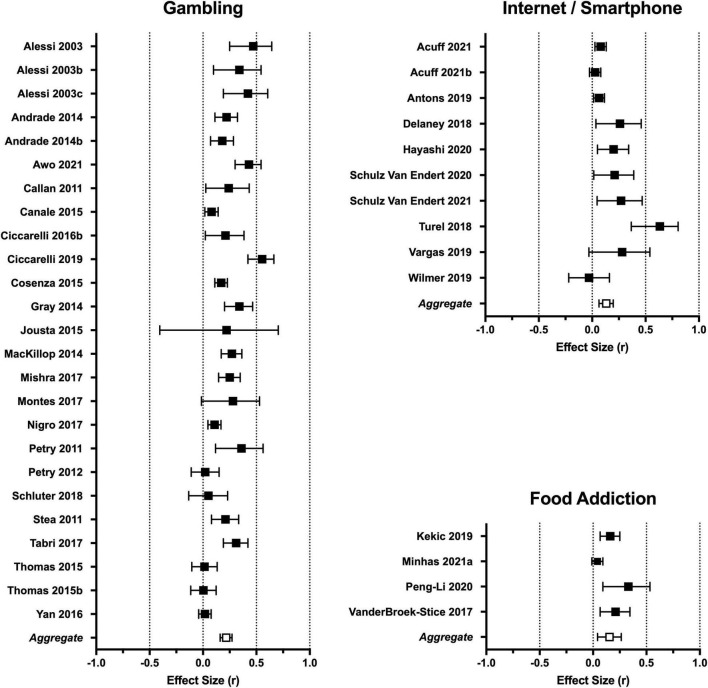
Forest plots depicting effect sizes for dimensional studies. Individual data points reflect effect size (Pearson’s r) and 95% confidence intervals. The aggregate effect size generated by the random-effects model is provided at the bottom of each forest plot. Complete data is provided in Supplementary Table 3.

Results of the aggregate and subgroup meta-analyses for categorical and dimensional studies are presented in Table 1 and effect sizes by study are presented in forest plots (Figures 2, 3). See Supplementary Table 3 for complete effect size data by individual studies.

**TABLE 1:** Meta-analytic results.

Category	*k*	*N*	*d* or *r*	*p*	95% CI	OSR	*Q*	*P*_*q*_	*I* ^2^
**Categorical designs**									
*Aggregate effect*	47	5,393	0.76	<0.0001	0.58–0.93	0.70–0.78	268.27	<0.0001	82.85
Gambling	28	2,252	0.82	<0.0001	0.60–1.04	0.76–0.88	113.02	<0.0001	76.11
Internet gaming disorder	13	641	0.89	<0.0001	0.53–1.24	0.68–0.94	65.76	<0.0001	81.75
Internet smartphone	6	2,500	0.16	0.141	−0.05–0.37	0.02–0.28	10.54	0.061	52.57
**Dimensional designs**									
*Aggregate effect*	40	13,441	0.19	<0.0001	0.15–0.23	0.18–0.20	198.94	<0.0001	80.40
Gambling	25	7,129	0.22	<0.0001	0.16–0.27	0.20–0.23	10.40	<0.0001	75.64
Internet smartphone	10	3,479	0.13	0.0001	0.06–0.20	0.10–0.16	10.40	0.0006	81.92
Food addiction	5	2,833	0.12	0.003	0.04–0.20	0.12–0.19	13.11	0.011	63.48

*k, # of effect sizes; N, total number of unique individuals; d, Cohen’s d effect size statistic for categorical designs; r, Pearson’s correlation coefficient for dimensional designs; p, statistical significance of effect size; OSR, range of effect sizes obtained from one-study-removed jackknife analysis; Heterogeneity statistics from the fixed effects analysis: Q, Cochran’s Q-test of homogeneity; P_q_, p-value corresponding to Cochran’s Q; I^2^, proportion of variability due to heterogeneity.*

### Categorical Studies

The aggregate meta-analysis for categorical studies included 47 effect sizes yielding an overall Cohen’s *d* of 0.76 (*p* < 0.0001), reflecting a medium-to-large effect size difference in discounting between the behavioral addiction groups and control groups. Results of the one-study-removed analysis revealed that no single study had a disproportionate impact on the aggregate effect size. There was substantial heterogeneity in the aggregate analysis, as indicated by both Cochran’s Q and I^2^ statistics (Table 1). Gambling, IGD, and internet/smartphone categories had sufficient effect sizes for subgroup analyses (findings of food addiction and compulsive buying categories are described in narrative review below). Gambling and IGD yielded comparable aggregate effect sizes (*d*s = 0.82 and 0.89, respectively) that were highly significant (*p*s < 0.0001; see Figure 2). However, the one-study-removed analysis revealed that the IGD category was markedly influenced by a single study (Raiha et al., 2020). Removal of this study reduced the aggregate effect size from *d* = 0.82 to 0.59. Both categories had statistically significant heterogeneity (see Table 1). In contrast to the findings for gambling and IGD, the aggregate effect size for internet/smartphone studies was small in magnitude and not statistically significant (*d* = 0.16, *p* = 0.141).

### Dimensional Designs

Before presenting the results for dimensional designs, an important detail to consider when aggregating correlations across studies is whether the sample was restricted to participants meeting clinical criteria or an established cutoff (e.g., participants diagnosed with gambling disorder or reporting a history of gambling problems) or a non-restricted sample of participants (i.e., a general sample of community volunteers or university students). The latter sample type presumably represents the full range of possible scores on the addiction scales, while the former may be subject to restricted range on the scales. All studies within the internet/smartphone and food addiction categories were non-restricted/general samples; eight of the 25 gambling studies were restricted to participants meeting clinical criteria for pathological gambling, gambling disorder, or reporting problems with gambling (see Supplementary Table 3).

The aggregate analysis of studies using dimensional designs included 40 effect sizes. The overall correlation across studies was small magnitude (*r* = 0.19, *p* < 0.0001). The one-study-removed analysis indicated minimal influence of individual studies on the overall effect size (*r* 0.18–0.20). Cochran’s Q and I^2^ statistics indicated substantial heterogeneity across studies (Table 1). Gambling, internet/smartphone, and food addiction had sufficient effect sizes for subgroup analysis (IGD and compulsive buying are summarized below). The effect size for gambling studies (*r* = 0.22) was moderately larger than the other two categories (*r* 0.12–0.13), with the caveat that all effect sizes are considered small magnitude (see Figure 3). As with the aggregate analysis, there was significant heterogeneity within each category (Table 1).

### Delay Discounting Measure Type

A moderator analysis was conducted to examine differences in effect size between types of delay discounting measures. Following a similar procedure as previous meta-analyses (e.g., Amlung et al., 2017), individual effect sizes were coded as either using the MCQ or a multi-item DDT. This latter category was considerably heterogeneous; however, there were insufficient studies with specific types of discounting tasks (e.g., adjusting amount vs. titration vs. experiential) to examine these individually. Therefore, the moderator analysis considered whether the MCQ yielded significantly different effect sizes compared to other “non-MCQ” discounting measures. At the aggregate level collapsing across all behavioral addiction types, there were no significant differences between MCQ and non-MCQ for categorical studies (MCQ *d* = 0.64, *k* = 21; non-MCQ *d* = 0.85, *k* = 26; *Q* = 1.43, *p* = 0.233) or dimensional studies (MCQ *r* = 0.19, *k* = 18; non-MCQ *r* = 0.19, *k* = 21; *Q* = 0.05, *p* = 0.819). Importantly, although the Cohen’s *d* for categorical studies was somewhat larger than MCQ studies, the between-study heterogeneity statistic was non-significant. There were also no significant differences between MCQ and non-MCQ measures when behavioral addiction types were examined separately (*p*s = 0.23–0.95). Thus, the moderator analysis provided evidence of similar effect sizes regardless of the type of discounting measure administered.

### Publication Bias

Publication bias indices were examined for two categorical design categories (gambling and IGD) and two dimensional design categories (gambling and internet/smartphone). Results are provided in Table 2. Owrin’s modified fail safe *N*-values for gambling categorical and dimensional studies indicated that 30 and 26 non-significant studies (respectively) would be needed to reduce the aggregate effect size by 50%. A smaller number of studies would be needed for IGD categorical (*k* = 13) and internet/smartphone dimensional (*k* = 11) to yield a similar 50% reduction. Kendall’s tau and Egger’s intercepts were significant for all but one category (gambling dimensional). The trim and fill approach identified missing effect sizes for the gambling and internet/smartphone categories (see funnel plots in Supplementary Figure 1). After imputation, the adjusted effect size was reduced for both categories (gambling: 0.22–0.16 and 0.13–0.08 for internet/smartphone). Of note, the lower bound of 95% confidence intervals for the adjusted internet/smartphone category approached 0.0, essentially indicating a non-significant aggregate effect size.

**TABLE 2:** Publication bias indices.

Category	Orwin’s *N*	Kendall’s tau	Egger’s intercept	Trim and fill # studies	Adjusted effect (CI)
**Categorical designs**					
Gambling	29	0.29*	4.21*	0	–
Internet gaming disorder	13	0.45*	6.28*	0	–
**Dimensional designs**					
Gambling	26	0.27	2.73*	6	0.16 (0.10–0.22)
Internet smartphone	11	0.49*	2.16*	4	0.08 (0.01–0.15)

Orwin’s N, Orwin’s modified fail-safe N assuming a 50% reduction in effect size. *Statistical significance (p < 0.05) of Kendall’s Tau (two-tailed) and Egger’s Intercept (one-tailed). CI, 95% confidence interval; Publication bias indices were not calculated for categories with less than 10 effect sizes (see Supplementary Table 3).

### Narrative Review of Studies Not in Meta-Analysis

Compulsive and pathological buying was the focus of only two included studies. Nicolai and Moshagen (2017) compared rates of delay discounting using AUC (for which greater values indicate a larger area, thus, less steep discounting) with severity of pathological buying using the pathological buying scale (PBS). The resulting correlation was −0.15, indicating that greater delay discounting was associated with increased severity on the PBS. Williams (2012) used a two-choice impulsivity program (TCIP) to examine discounting between a group of healthy controls and a group of individuals who met the proposed DSM criteria for impulse control disorder (ICD) for compulsive buying. Taking the sum of impulsive choices across groups, the mean and standard deviation for the compulsive buying group was 20.56 (13.82), and for the healthy controls group was 8.5 (9.33). Thus, individuals in the compulsive buying group selected more immediate choices on the TCIP than individuals in the control group.

In addition to the four dimensional food addiction studies included in the meta-analysis (Davis et al., 2011), employed a categorical design comparing individuals who met the Yale Food Addiction Scale (YFAS) diagnostic scoring criteria for food addiction to a group of controls. The mean and standard deviation of indifference points for the food addiction group was 231.7 (138.2) and for the control group was 306.5 (123.2), indicating that individuals in the food addiction group generally had steeper delay discounting rates than controls.

Two studies focusing on IGD employed dimensional designs. Acuff et al. (2021) correlated delay discounting (using ICR) with severity of responses on the Gaming Addiction Scale (GAS). The resulting Pearson *r* correlation was 0.031. Bailey et al. (2013) also correlated ICR with severity responses on a revised version of the Problematic Video Game Play (PVP) Scale, reporting a correlation of 0.12.

## Discussion

The results of this meta-analysis show that individuals across a range of behavioral addictions exhibit similar patterns of steeper delay discounting both compared to controls and as a function of the severity of the behavioral addiction. We found statistically significant results for the two aggregate analyses and significant effects for most behavioral addictions categories. However, several categories returned larger effect sizes than others. The effect sizes from the analysis of categorical studies in gambling and IGD categories were medium-to-large magnitude (although IGD was strongly influenced by a single study), while the effect size for internet/smartphone addiction was smaller and not statistically significant. The effect sizes from the analysis of continuous measures returned a somewhat different pattern of results. Although the category-specific effect sizes for gambling, food, and internet/smartphone addiction were statistically significant, the aggregate correlation for gambling was larger than for internet/smartphone or food addiction. One possible explanation for these discrepancies is that the scales used in the internet/smartphone addiction studies require additional validation and perhaps are not identifying certain behavior patterns that more well-validated scales, such as those for gambling and IGD, can ascertain. Due to the current ubiquity of mobile devices, additional scale validation and delay discounting research in this area is warranted.

Gambling disorder has been a category of focus in two prior meta-analyses (MacKillop et al., 2011; Amlung et al., 2017). Synthesizing research on continuous associations, Amlung et al. (2017) calculated a Pearson *r* effect size statistic of 0.16. In the current meta-analysis, the overall effect size statistic of gambling studies using dimensional designs was slightly larger (*r* = 0.22). MacKillop et al. (2011) calculated effect size statistics for studies with categorical designs. The overall effect size for the clinical group was 0.79, and for the subclinical group was 0.41, whereas the Cohen’s *d* in the current meta-analysis was 0.82. The number of gambling disorder studies using categorical designs increased from 7 total in MacKillop et al., 2011 to 28 studies in the current analysis. A smaller number of dimensional studies were added (4 more than Amlung et al., 2017); however, the change in total sample size was substantial, increasing from 2,940 to 7,129. It is plausible that modest increase in aggregate effect size was due, in part, to greater precision from larger sample sizes. In sum, the addition of updated gambling studies results in somewhat larger effect sizes for both dimensional and categorical designs.

The relationship between delay discounting rate and presence of IGD has been a focus of prior meta-analyses (Cheng et al., 2021; Yao et al., 2021). Cheng et al. (2021) focused only on categorical designs. While the present review was originally designed to examine dimensional designs, there were not enough to be included in the meta-analysis. Cheng et al. (2021) calculated an overall effect size statistic for studies that used *k*-values to analyze discounting rate (*k*) of Hedges’ *g* = 0.76, and for studies that used AUC of *g* = 1.44. Similarly, Yao et al. (2021) included categorical designs but also focused on a range of decision-making deficits beyond discounting. The effect size statistic (*g*) for delay discounting was 0.58 while *d* = 0.68 in our analysis after removal of the highly influential result from Raiha et al., 2020. Both results indicate steeper discounting in participants with IGD. Updating past meta-analyses with sufficient new research advances our understanding of the relationship between delay discounting and the present disorders. Indeed, we found that recent research has further strengthened the relationship between gambling disorder and IGD and steep delay discounting.

Food addiction and obesity, while occasionally conflated, are in fact distinctly separate constructs (Gordon et al., 2018). Thus, the results from the food addiction category should not be compared to the results of the meta-analysis on delay discounting among individuals with obesity conducted by Amlung et al. (2016). Indeed, apart from Davis et al. (2011) in which the inclusion criteria for participation was a body mass index (BMI) in the obese range, the average BMI in most studies in the food addiction category was in the normal weight range. While a positive association was found across most studies between food addiction severity and BMI, this was not the focus of the present review and future research examining the relationship between BMI, food addiction, and delay discounting is warranted.

The paucity of compulsive or pathological buying studies, food addiction studies using categorical designs, and IGD studies using dimensional designs prevented us from calculating aggregate effect sizes. While the narrative summary of these studies generally suggests steeper discounting associated with presence of these behavioral addictions, we are unable to evaluate the reliability of these findings or directly compare the results to the other categories included in the meta-analysis. Replications and extensions of the current research in these areas is integral. Additionally, it is worth highlighting that in the search for studies to include in the current systematic review and meta-analysis, several categories proposed as behavioral addictions (e.g., sex, love, work, indoor tanning, kleptomania) returned no results (see Supplementary Table 1). These too are areas of importance for future research.

The current review and meta-analysis raises several additional questions for future research. First, because not all categories included in the current review are officially recognized as behavioral addictions, whether some of these categories are over-pathologized should be a topic of continued research and discourse (Billieux et al., 2015). By definition, “impulsivity” inherently pathologizes behavior patterns that may not necessarily be maladaptive. Such a concern can be raised for all behaviors labeled “impulsive.” A functional and theoretical approach to describing behavior patterns often characterized as facets of impulsivity—specifically, steep delay discounting—is integral to our understanding of the importance of and limitations to this line of research.

The considerable heterogeneity across studies within each category limits the generality of these findings. While the use of a random-effects model addresses this limitation to some extent, the differences in the discounting tasks and behavioral addiction scales used may have impacted the results. Additionally, while we did not include groups of subjects for whom there was an explicit comorbid substance use or other psychiatric disorder, we did not exclude all studies in which there were possible comorbidities with behavioral addictions. Many behavioral addictions likely include co-morbidity with substance use disorders which may be difficult or impossible to disentangle based on participant descriptions, thus, these comorbidities may have confounded the results in unknown ways.

We could not always identify the specific procedures of the DDTs. However, the results of the moderator analysis indicated similar effect sizes in studies using the MCQ compared to non-MCQ measures. This is consistent with prior meta-analyses reporting no significant differences between MCQ and non-MCQ multi-item tasks (e.g., MacKillop et al., 2011; Amlung et al., 2017). Providing details of delay discounting methods in future studies may help in determining whether more specific types of discounting task used may function as a moderator between discounting rates and the independent variable of interest. Though the exact task procedures in the included studies were not always clear, it is worth discussing the potential implications of the use of monetary rewards as the only target commodity for all included studies except one (Buono et al., 2017). Individuals with substance use disorder tend to discount their substance of choice (e.g., cigarettes, crack/cocaine, cannabis, alcohol) more steeply than monetary rewards (e.g., Bickel et al., 1999; Coffey et al., 2003; Johnson et al., 2010; Moody et al., 2017). Buono et al. (2017) examined discounting of monetary rewards and video game time among high-, medium-, and low-frequency video game players. Results indicated that AUC was lower (i.e., steeper discounting) across all groups when the commodity was video game play compared to money. Although these findings are from a single study, they do underscore the need for additional investigation of commodity effects in behavioral addictions.

To our knowledge, this is the first systematic review and meta-analysis comparing rates of delay discounting across multiple categories of behavioral addictions. In sum, the results revealed that there is generally a relationship between steepness of delay discounting rates and severity of behavioral addiction (except for internet/smartphone addiction); however, the magnitude of these relationships varies across categories. Several categories included in the review are not listed as addictions in the DSM-5 (food addiction, internet/smartphone addiction, compulsive/pathological buying) and thus warrant caution when interpreting results. Additionally, some scales used to assess the presence and severity of a given behavioral addiction are not as well-validated as others, which may have contributed to the smaller effect sizes in the internet/smartphone category. Importantly, the present review highlights the need for additional research to deepen our understanding of the relationship between discounting and behavioral addiction.

## Data Availability Statement

The original contributions presented in the study are included in the article/Supplementary Material, further inquiries can be directed to the corresponding author/s.

## Author Contributions

MA, SB, and IB: study conception. SW and MA: study selection, data analysis, and manuscript development. SW: data extraction. IB, SB, and LM: manuscript edits and feedback. All authors: approval of final manuscript.

## Conflict of Interest

The authors declare that the research was conducted in the absence of any commercial or financial relationships that could be construed as a potential conflict of interest.

## Publisher’s Note

All claims expressed in this article are solely those of the authors and do not necessarily represent those of their affiliated organizations, or those of the publisher, the editors and the reviewers. Any product that may be evaluated in this article, or claim that may be made by its manufacturer, is not guaranteed or endorsed by the publisher.

## Footnotes

1
www.covidence.org

2
https://apps.automeris.io/wpd/
