# Supplementary material for: Delay Discounting in Established and Proposed Behavioral Addictions: A Systematic Review and Meta-Analysis
Source: Front Behav Neurosci. 2021 Nov 26;15:786358. doi: 10.3389/fnbeh.2021.786358 (PMC8661136; doi:10.3389/fnbeh.2021.786358)

**SUPPLEMENTARY MATERIALS**

**Supplemental Table 1**

*Search Term Strategy*

| Search number | Query | Total # Results Returned |
| --- | --- | --- |
| 1 | "discounting" | 8,908 |
| 2 | "gambling" | 23,175 |
| 3 | #1 AND #2 | 554 |
| 4 | "sexu*" | 548,598 |
| 5 | #1 AND #4 | 267 |
| 6 | "gaming disorder" | 1,680 |
| 7 | "gaming addict*" | 489 |
| 8 | "compulsive overeating" | 172 |
| 9 | "hyperphagia" | 8,381 |
| 10 | "uncontrolled eating" | 382 |
| 11 | "food addiction" | 4,218 |
| 13 | #1 AND #11 | 18 |
| 14 | #1 AND #10 | 4 |
| 15 | #1 AND #9 | 16 |
| 16 | #1 AND #8 | 4 |
| 17 | #1 AND #6 | 32 |
| 18 | #1 AND #7 | 6 |
| 19 | internet use disorder | 44,261 |
| 20 | "internet addiction" | 5,391 |
| 21 | "exercis*" | 541,826 |
| 22 | "tanning" | 4,161 |
| 23 | "love" | 50,286 |
| 24 | "smartphone*" | 21,376 |
| 25 | social media | 111,396 |
| 26 | "problem spending" | 3 |
| 27 | "compulsive buying" | 937 |
| 28 | "compulsive shop*" | 395 |
| 29 | "workahol*" | 900 |
| 30 | "work addict*" | 406 |
| 31 | #1 AND #19 | 146 |
| 32 | #1 AND #20 | 25 |
| 33 | #1 AND #21 | 164 |
| 34 | #1 AND #22 | 4 |
| 35 | #1 AND #23 | 14 |
| 36 | #1 AND #24 | 28 |
| 37 | #1 AND #25 | 92 |
| 38 | #1 AND #26 | 2 |
| 39 | #1 AND #27 | 3 |
| 40 | #1 AND #28 | 5 |
| 41 | #1 AND #29 | 1 |
| 42 | #1 AND #30 | 0 |
| 43 | food addiction | 6,777 |
| 44 | #1 AND #43 | 95 |
| 45 | "klepto*" | 992 |
| 46 | #1 AND #46 | 0 |

Note. Searches in which the term ends in an asterisk are “wild card” searches, meaning that all possible endings to that word are included in the search.

**Supplementary Table 2.**

*Demographic Information and Characteristics of Included Studies*

| **Prev.**  **MA?** | **Reference** | **N total** | **DRD Measure** | **DRD Commodity** | **Hypothetical/ Actual Rewards?** | **DRD Index** | **Diagnostic Tool/Scale** | **Age - M(SD) or range** | **% Female** | **Race / Ethnicity** |  |
| --- | --- | --- | --- | --- | --- | --- | --- | --- | --- | --- | --- |
|  | **Compulsive Pathological Buying** | | | | | | | | | | |
|  | Williams, 2012 | 53 | TCIP | Money | Not reported | Total immediate choices | DSM-IV Compulsive buying | Addiction group: 28.3 (11.5); Control: 28.4 (5.9) | 83% | Not reported |  |
|  | Nicolai & Moshagen, 2017 | 103 | DDT – Adjusting Amount; Du et al. 2002. | Money | Hypothetical | AUC | PBS | 28.2 (9.4) | 70% | Not reported |  |
|  | **Food Addiction** | | | | | | | | | | |
|  | Minhas et al., 2021a | 729 | MCQ | Money | Not reported | log(k) | YFAS | 21.4(1.2) | Not reported | White/European (71.2%) |  |
|  | Minhas et al., 2021b | 1427 | MCQ | Money | Not reported | log(k) | YFAS | 38.9 (13.7) | 58% | White/European (81.8%) |  |
|  | Peng-Li et al., 2020 | 64 | DDT – Adjusting Amount; Du et al. 2002 | Money | Actual | Not reported | YFAS | 21.5 (0.9) | 69% | Not reported |  |
|  | Davis et. al, 2011 | 72 | DDT – Adjusting Amount; Richards, et al. 1997 | Money | Hypothetical | Indifference point | YFAS | 25-46 | 68% | Not reported |  |
|  | Kekic et al., 2019 | 432 | DDT – Adjusting Amount and Delay; Steinglass et al., 2012 | Money | Hypothetical | Discounting factor (DF) | YFAS | 18-65+ | 89% | Arab (2.8); Asian (4.6%); Black (6.7%); Mixed (10.2%); White (70.4%); other (2.8%) |  |
|  | VanderBroek-Stice et al., 2017 | 181 | MCQ and DDT; Composite variable used; Amlung et al., 2014 | Money | Hypothetical | log(k) | YFAS | 24.8 (9.5) | 71% | American Indian/Alaskan Native (0.6%); Asian/Pacific Islander (7.7%); Black/African American (23.2%); Mixed (5.5%); White (63%) |  |
|  | **Gambling** | | | | | | | | | | |
| A | Albein-Urios et. al, 2012 | 43 | MCQ | Money | Hypothetical | AUC | DSM-IV | 35.6 (8.7) | Not reported | Not reported |  |
|  | Albein-Urios et. al, 2014 | 64 | MCQ | Money | Hypothetical | k | DSM-IV | 19-52 | Not reported | Not reported |  |
| A | Alessi & Petry, 2003 | 62 | DDT – Index Cards; Petry & Casarella, 1999 | Money | Hypothetical | k | ASI-G, SOGS, DSM-IV criteria | 44 (11) | 32% | African American (10%); Caucasian (84%); other (6%) |  |
| A | Andrade et. al, 2014 | 315 | MCQ | Money | Hypothetical | k | ASI-G, SOGS | 40.8 (SD not reported) | 42% | African American (39%); Hispanic (18%); White (43%) |  |
|  | Awo et. al, 2021 | 174 | Single-shot | Money | Hypothetical | DDS score | PGSI | 21-34 | 8% | Not reported |  |
|  | Brevers et al., 2017 | 62 | DDT – Adjusting Amount; Madden et al., 1997 | Money | Hypothetical | Indifference point | DSM-IV | Gambling: 40.2 (10.2); Control: 44.1 (11.0) | 23% | Not reported |  |
| A | Callan et. al, 2011 | 83 | DDT – Adjusting Amount; No Citation Given | Money | Hypothetical | AUC | PGSI | 20-78 | 49% | Not reported |  |
|  | Calluso et. al, 2020 | 47 | DDT – Intertemporal Choice Task; Calluso et al. 2015 | Money | Hypothetical | log(k) | SOGS | Gambling: 45.2 (11.5); control: 42.8 (8.2) | 11% | Not reported |  |
| A | Canale et. al, 2015 | 986 | MCQ | Money | Hypothetical | ICR | SOGS-RA | 19.5 (2.3) | 36% | Not reported |  |
|  | Ciccarelli et. al, 2016 | 108 | MCQ | Money | Hypothetical | k | SOGS | 24-65 | 0% | Not reported |  |
|  | Ciccarelli et. al, 2019 | 128 | MCQ | Money | Hypothetical | k | SOGS | 18-67 | 27% | Not reported |  |
|  | Contrereas-Rodriguez et. al, 2015 | 40 | MCQ | Money | Hypothetical | k | DSM-IV | 32.4 (SD not reported) | 8% | Not reported |  |
|  | Cosenza et. al, 2015 | 1039 | MCQ | Money | Hypothetical | ln(k) | SCI-PG | 16-19 | 49% | Not reported |  |
|  | Cosenza et. al, 2017 | 104 | MCQ | Money | Hypothetical | ln(k) | SOGS-RA | 16-19 | 0% | Not reported |  |
| A | DeWilde et. al, 2013 | 53 | DDT – Adjusting Amount; Richards et al., 1999 | Money | Hypothetical | k | SOGS, SCID-I | 30.3 (SD not reported) | 11% | Not reported |  |
| M | Dixon et. al, 2003 | 40 | MCQ | Money | Hypothetical | AUC | SOGS | 40.6 (SD not reported) | 30% | Not reported |  |
|  | Freinhofer et. al, 2020 | 49 | DDT – Intertemporal Choice Task; Pine et al. 2009 | Money | Hypothetical | ICR | SOGS | Gambling: 43.9 (11.9); Control: 40.8 (14.3) | 12% | Not reported |  |
| A | Gray et. al, 2014 | 175 | MCQ | Money | Hypothetical | k | SCI-PG | 34.7 (13.1) | 13% | Not reported |  |
|  | Hiraoka, 2018 | 14 | DDT – Index Card Task; Sugiwaka & Okouchi, 2004 | Money | Hypothetical | k | SOGS | Gambling: 21.4 (1.8); Control; 20.6 (1.1) | 36% | Not reported |  |
| M | Holt et. al, 2003 | 38 | DDT – Adjusting Amount; No Citation Given | Money | Hypothetical | AUC | SOGS | 18-24 | 32% | Not reported |  |
| A | Joutsa et al., 2015 | 24 | MCQ | Money | Hypothetical | k | DSM-IV criteria | 18-73 | Not reported | Not reported |  |
|  | Kraplin et al., 2014 | 38 | DDT – Intertemporal Choice Task; No Citation Given | Money | Hypothetical | k | DSM-IV criteria | 18-73 | Not reported | Not reported |  |
| M | Ledgerwood et al., 2009 | 71 | DDT – Intertemporal Choice Task; Petry & Casarella (1999) | Money | Hypothetical | AUC | NODS | Gambling: 48.4 (14.0); Control: 45.7 (17.9) | Gambling: 53%; Control: 59% | Gambling: Caucasian (86.7%); other (13.3%); Control: Caucasian (87.8%); other (12.2%) |  |
| M | MacKillop et al., 2006 | 93 | DDT – Adjusting Amount Madden et al. 1997 | Money | Not reported | log(k) | SOGS | 19.5 (SD not reported) | 25% | African American (6.5%); Asian (30.6%); Caucasian (54.8%); Latino (8.1%) |  |
| A | MacKillop et al., 2014 | 353 | MCQ | Money | Actual | k | SCI-PG | 35.3 (2.3) | 22% | White (52.1%), African American (43.1%), Mixed race (2.8), Asian (1.1%), N/A (.6%), Other (.3%), Hispanic (1.4%) |  |
| A, M | Madden et al., 2009 | 39 | MCQ | Money | Hypothetical | log(k) | SOGS | Gambling: 37.7 (2.4); Control: 37.2 (2.2) | 0% | African American (36.8%); Caucasian (47.4%), other (15.8%) |  |
|  | Michalczuk et al., 2011 | 57 | MCQ | Money | Hypothetical | ln(k) | MAGS | Gambling: 40.1 (12.3); Control: 35.8 (12.2) | 6% | Not reported |  |
|  | Miedl et al., 2012 | 32 | DDT – Intertemporal choice task; Peters & Buchel, 2009 | Money | Actual | k | SOGS | Gambling: 35 (2); Control: 38 (2) | 6% | Not reported |  |
|  | Miedl et al., 2015 | 30 | MCQ | Money | Actual | log(k) | DSM-IV | Gambling: 36.7 (5.8); Control: 36.8 (5.6) | 0% | Not reported |  |
|  | Mishra et al., 2017 | 328 | MCQ | Money | Actual | k | PGSI | 31 (12.5) | 49% | Not reported |  |
|  | Mohammadi et al., 2016 | 30 | MCQ | Money | Actual | k | SOGS | Gambling: 36.7 (5.8); Control: 36.8 (5.6) | 0% | Not reported |  |
|  | Montes et al., 2017 | 45 | DDT – Fill-in-the-Blank Version; Chapman, 1996 | Money | Hypothetical | AUC | SOGS | Gambling: 22.4 (7.4); Control: 19.5 (1.35) | 40% | Gambling: Non-White (4.44%); White (37.7%); Control: Non-White (4.44%); White (53.3%). |  |
|  | Nigro & Cosenza, 2016 | 138 | MCQ | Money | Hypothetical | k | SOGS-RA | 17.6 (0.7) | 20% | Not reported |  |
|  | Nigro et al., 2017 | 1,010 | MCQ | Money | Hypothetical | k | SOGS-RA | 15.4 (2.1) | 53% | Not reported |  |
| A | Petry, 2001 | 60 | DDT – Index Cards; Petry & Casarella 1999 | Money | Hypothetical | k | SOGS | Not reported | 33% | Not reported |  |
| A | Petry, 2012 | 226 | MCQ | Money | Actual | k | SOGS | 44.8 (10.9) | 44% | African American (8.4%); Hispanic (4.4%); White (84.5%); other (2.7%) |  |
|  | Schluter et al., 2018 | 116 | DDT – Adjusting Amount; Richards et al. 1999 | Money | Hypothetical | AUC | NODS | 37.6 (11.4) for Part 1; Age N/A for part 2 | 46% | Arab (0.9%); Black (10%); Caucasian (79.1%); Chinese (1.8%); Korean (0.9%); Latin American (12.7%); Southeast Asian (0.9%) |  |
|  | Secades-villa et al., 2016 | 874 | DDT – Adjusting Amount; Holt et al. 2012 | Money | Hypothetical | log(k) | SOGS-RA | Gambling: 14 (0.66); Control: 14.9 (0.5) | Gambling: 24%; Control: 45% | Not reported |  |
| A | Stea et al., 2011 | 218 | DDT – Adjusting Amount; Holt et al. 2003 | Money | Hypothetical | AUC | PGSI | 21.3 (4.4) | 63% | Caucasian (43.1%); Asian (39.9%); other (17.0%) |  |
|  | Tabri et al., 2017 | 239 | MCQ | Money | Hypothetical | ICR | PGSI | 36.7 (11.3) | 44% | Not reported |  |
| A | Thomas et al., 2015 | 272 | DDT – Multiple Choice Method Beck & Triplett, 2009 | Money | Hypothetical | AUC | SOGS | 20 (3.1) | 76% | Caucasian (91.5%) |  |
|  | Torres et al., 2013 | 44 | MCQ | Money | Hypothetical | Total immediate choices | DSM-IV | Gambling: 31.4 (5.9); Control: 30.1 (8.6) | Gambling: 10%; Control: 9% | Not reported |  |
|  | Weidacker et al., 2020 | 26 | MCQ | Money | Hypothetical | ln(k) | PGSI, SOGS, DSM-V | Gambling: 36.3 (9.5); Control: 35.7 (8.7) | 0% | GD: 97% White; Control: 100% White |  |
|  | Wiehler et al., 2015 | 40 | MCQ | Money | Hypothetical | log(k) | SOGS, KFG | 18-59 | 5% | Not reported |  |
|  | Williams, 2012 | 49 | TCIP | Money | Not reported | Total immediate choices | SOGS | Gambling: 39.4 (11.8); Control: 28.4 (5.9) | 57% | Not reported |  |
|  | Wolfling et al., 2020 | 58 | DDT – Adjusting Amount; Richards et al., 1999 | Money | Hypothetical | AUC | BIG-S | Gambling: 35.0 (11.4); Control: 25.6 (3.3) | Not reported | Not reported |  |
|  | Yan et al., 2016 | 1120 | MCQ | Money | Hypothetical | log (k) | SOGS | 18-23 | 71% | 62% Hans |  |
|  | **Internet Gaming Disorder** | | | | | | | | | | |
|  | Acuff et al., 2021 | 1406 | DDT – Intertemporal Choice Task; Gray et al. 2014 | Money | Hypothetical | ICR | GAS | 20.9 (3.7) | 74% | Not reported |  |
|  | Bailey et. al, 2013 | 149 | MCQ | Money | Hypothetical | ICR | Revised PVP scale | 16-30 | 47% | Not reported |  |
| C | Buono et. al, 2017 | 104 | MCQ | Money; Video game play time | Hypothetical | AUC | Frequency of video game play | 24.9 (5.1) | 36% | Asian Indian (6%); Asian / Pacific Islander (8%); Hispanic (6%); White (82%); other (6%) |  |
| C | Irvine et al., 2013 | 52 | MCQ | Money | Hypothetical | k | GAS | IGD: 24.7 (5.9), Control: 25.6 (5.9) | 12% | Not reported |  |
|  | Raiha et al., 2020 | 63 | DDT – Adjusting Amount; No Citation Given | Money | Not reported | AUC | IAT | IGD: 22.1 (3.7); Control: 21.9 (3.5) | 0% | Not reported |  |
| C, Y | Tian et al., 2018 | 83 | DDT – Adjusting Amount; Du et al. 2002 | Money | Hypothetical | AUC | YDQ | IGD: 15.6 (1.2); Control: 15.8 (0.9) | IGD: 48%; Control: 49% | Not reported |  |
| C, Y | Wang et al., 2017a | 40 | DDT – Intertemporal Choice Task; No Citation Given | Money | Actual | log (k) | IAT | IGD: 20.9 (2.4); Control: 21.9 (2.5) | 0% | Not reported |  |
|  | Wang et al., 2017b | 39 | DDT – Intertemporal Choice Task; Wang et al. 2017a | Money | Actual | log (k) | IAT | IGD: 22.1 (3.2); Control: 23.1 (2.0) | 0% | Not reported |  |
|  | Wang et al., 2017c | 39 | DDT – Intertemporal Choice Task; Wang et 2017a | Money | Actual | log (k) | IAT | IGD: 22.1 (3.2); Control: 23.1 (2.0) | 0% | Not reported |  |
| C | Weinstein et al., 2016 | 40 | DDT – Adjusting Amount; Rachlin et al. 1991 | Money | Not reported | k | POGQ- short form | 22-28 | 5% | Not reported |  |
| C, Y | Wolfling et al., 2020 | 57 | DDT – Adjusting Amount; Richards et al. 1999 | Money | Hypothetical | AUC | AICA-C | IGD: 26.9 (5.9); Control: 25.6 (3.2) | Not reported | Not reported |  |
|  | Yan et al., 2021 | 115 | MCQ | Money | Hypothetical | log (k) | IAT | IGD: 20.2 (1.4); Control: 20.2 (1.4) | IGD: 40%; Control: 38% | IGD: Hans (72.4%); Control: Hans (69.8%) |  |
| Y | Yao et al., 2017 | 46 | MCQ | Money | Hypothetical | log (k) | DSM-5, CIAS | 18-26 | Not reported | Not reported |  |
|  | **Internet Smartphone** | | | | | | | | | | |
|  | Acuff et al., 2021 | 1406 | DDT – Intertemporal Choice Task; Gray et al. 2014. | Money | Hypothetical | ICR | PIUQ, SAS | 20.9 (3.7) | 74% | Not reported |  |
|  | Antons et. al, 2019 | 1498 | MCQ | Money | Hypothetical | ln(k) | IAT | 18-83 | 0% | Not reported |  |
|  | Delaney et. al, 2018 | 75 | DDT – Adjusting Amount; Rachlin et al., 1991 | Money | Hypothetical | k | BFAS | 19.8 | 52% | Not reported |  |
|  | Hayashi et. al, 2020 | 167 | DDT – Intertemporal Choice Task; Jones & Rachlin, 2009 | Money | Hypothetical | AUC | STDS | 19.9 (4.1) | 57% | Not reported |  |
|  | Hayashi et. al, 2021 | 107 | DDT – Intertemporal Choice Task; Rachlin et al. 1991 | Money | Hypothetical | AUC | TIC | 19.35 | 59% | Not reported |  |
| C | Li et al., 2016 | 56 | MCQ | Money | Hypothetical | AUC | YDQ | PIU: 21.0 (1.7); Control: 21.1 (1.6) | 58.9% | Not reported |  |
| C | Liu et al., 2019 | Sample 1=1,281 Sample 2=1,034 | MCQ | Money | Not reported | log(k) | IAT | Sample 1 = 19.1 (1.1); Sample 2 = 19.2 (1.1) | Sample 1=66%; Sample 2=61% | Not reported |  |
| C | Savillle et al., 2010 | 28 | DDT – Intertemporal Choice Task; Rachlin et al. 1991 | Money | Hypothetical | k | IAT | Addiction: 19.2 (1.0); Control: 19.1 (1.1) | 85.7% | Not reported |  |
|  | Schulz Van Endert, 2021 | 75 | MCQ | Money | Hypothetical | ln(k) | DASC | 11.3 (SD not reported) | 47% |  |  |
|  | Schulz Van Endert, 2020 | 101 | MCQ | Money | Hypothetical | ln(k) | Study specific scale | 22 (SD not reported) | 52% | Not reported |  |
|  | Turel et al., 2018 | 32 | DDT – Intertemporal Choice task; Wang et al. (2014) | Money | Not reported | k | Study specific interview | 31.2 (9.3) | 19% | Not reported |  |
| C | Vargas et al., 2019 | 41 | DDT – Adjusting Amount; Pornpattananangkul et al., 2017 | Money | Hypothetical | k | PIUQ | PIU: 18.8 (1.1); Control: 19.2 (1.2) | 58% | Not reported |  |
|  | Wilmer et al., 2019 | 106 | DDT – Adjusting Amount; O’Brien et al. 2001 | Money | Hypothetical | log(k) | MMPUS | 20.1 (2.5) | 70% | African American/Black (13.2%); Asian (10.4%); Caucasian/White (67.9%); more than one race (5.7%), declined to respond (2.8%) |  |

*Note*.

Studies included in previous meta-analyses are indicated in the “Prev. MA” column (M = MacKillop et al. 2011; A = Amlung et al. 2017; C = Cheng et al. 2021; Y = Yao et al. 2021

Abbreviations:

TCIP = two-choice impulsivity paradigm. DDT = delay discounting task. MCQ = monetary choice questionnaire. SOGS = South Oaks Gambling Screen. PBS = Pathological Buying Screener. YFAS = Yale Food Addiction Scale. BES = Binge Eating Scale. EAT-26 = Eating Attitudes Test. ASI-G = Addiction Severity Index - Gambling. PGSI = Problem Gambling Severity Index. SOGS – RA = South Oaks Gambling Screen – Revised for Adolescents. SCI - PG = Structured Clinical Interview for Pathological Gambling. SCID – I = Structured Clinical Interview for DSM Axis I Disorders. NODS = The NORC Diagnostic Screen for Gambling Problems. MAGS = Massachusetts Gambling Screen. KFG = Kurzfragebogen zum Glücksspielverhalten. BIG – S = Berlin Inverntory of Gambling Behavior - Screening. GAS = Gaming Addiction Scale. PVP = Problem Video Game Playing Scale. IAT = Internet Addiction Test. YDQ = Young Diagnostic Questionnaire. POGQ = Problematic Online Gambling Questionnaire. AICA-C = Assessment of Internet and Computer Game Addiction. CIAS = Chen Internet Addiction Scale. PIUQ = Problematic Internet Use Questionnaire. SAS = Smartphone Addiction Scale. BFAS = Bergen Facebook Addiction Scale. STDS = Self-perception of Text Message Dependency Scale. TIC = texting in the classroom. DASC = Digital Addiction Scale for Children. SNS = social networking site. MMPUS = Mobile Phone Problem Use Scale.

References:

- Amlung, M., Sweet, L.H., Acker, J., Brown, C.L., and MacKillop, J. (2014). Dissociable brain signatures of choice conflict and immediate reward preferences in alcohol use disorders. *Addiction biology* 19(4)**,** 743-753. doi: 10.1111/adb.12017.
- Beck, R.C., and Triplett, M.F. (2009). Test–retest reliability of a group-administered paper–pencil measure of delay discounting. *Experimental and clinical psychopharmacology* 17(5)**,** 345. doi: 10.1037/a0017078.
- Calluso, C., Committeri, G., Pezzulo, G., Lepora, N., and Tosoni, A. (2015). Analysis of hand kinematics reveals inter-individual differences in intertemporal decision dynamics. *Experimental brain research* 233(12)**,** 3597-3611. doi: 10.1007/s00221-015-4427-1.
- Chapman, G.B. (1996). Temporal discounting and utility for health and money. *Journal of Experimental Psychology: Learning, Memory, and Cognition* 22(3)**,** 771. doi: 10.1037/0278-7393.22.3.771.
- Du, W., Green, L., and Myerson, J. (2002). Cross-cultural comparisons of discounting delayed and probabilistic rewards. *The Psychological Record* 52(4)**,** 479-492. doi: 10.1007/BF03395199.
- Gray, J.C., Amlung, M.T., Acker, J.D., Sweet, L.H., and MacKillop, J. (2014). Item-based analysis of delayed reward discounting decision making. *Behavioural processes* 103**,** 256-260. doi: 10.1016/j.beproc.2014.01.006.
- Holt, D.D., Green, L., and Myerson, J. (2003). Is discounting impulsive?: Evidence from temporal and probability discounting in gambling and non-gambling college students. *Behavioural processes* 64(3)**,** 355-367. doi: 10.1016/S0376-6357(03)00141-4.
- Holt, D.D., Green, L., and Myerson, J. (2012). Estimating the subjective value of future rewards: Comparison of adjusting-amount and adjusting-delay procedures. *Behavioural processes* 90(3)**,** 302-310. doi: 10.1016/j.beproc.2012.03.003.
- Jones, B.A., and Rachlin, H. (2009). Delay, probability, and social discounting in a public goods game. *Journal of the experimental analysis of behavior* 91(1)**,** 61-73. doi: 10.1901/jeab.2009.91-61.
- Madden, G.J., Petry, N.M., Badger, G.J., and Bickel, W.K. (1997). Impulsive and self-control choices in opioid-dependent patients and non-drug-using control patients: Drug and monetary rewards. *Experimental and clinical psychopharmacology* 5(3)**,** 256. doi: 10.1037/1064-1297.5.3.256.
- O'Brien, L., Albert, D., Chein, J., and Steinberg, L. (2011). Adolescents prefer more immediate rewards when in the presence of their peers. *Journal of Research on adolescence* 21(4)**,** 747-753. doi: 10.1111/j.1532-7795.2011.00738.x.
- Peters, J., and Büchel, C. (2009). Overlapping and distinct neural systems code for subjective value during intertemporal and risky decision making. *Journal of Neuroscience* 29(50)**,** 15727-15734. doi: 10.1523/JNEUROSCI.3489-09.2009.
- Petry, N.M., and Casarella, T. (1999). Excessive discounting of delayed rewards in substance abusers with gambling problems. *Drug and alcohol dependence* 56(1)**,** 25-32. doi: 10.1016/S0376-8716(99)00010-1.
- Pine, A., Seymour, B., Roiser, J.P., Bossaerts, P., Friston, K.J., Curran, H.V., et al. (2009). Encoding of marginal utility across time in the human brain. *Journal of Neuroscience* 29(30)**,** 9575-9581. doi: 10.1523/JNEUROSCI.1126-09.2009.
- Pornpattananangkul, N., Nadig, A., Heidinger, S., Walden, K., and Nusslock, R. (2017). Elevated outcome-anticipation and outcome-evaluation ERPs associated with a greater preference for larger-but-delayed rewards. *Cognitive, Affective, & Behavioral Neuroscience* 17(3)**,** 625-641. doi: 10.3758/s13415-017-0501-4.
- Rachlin, H., Raineri, A., and Cross, D. (1991). Subjective probability and delay. *Journal of the experimental analysis of behavior* 55(2)**,** 233-244. doi: 10.1901/jeab.1991.55-233.
- Richards, J.B., Mitchell, S.H., De Wit, H., and Seiden, L.S. (1997). Determination of discount functions in rats with an adjusting‐amount procedure. *Journal of the Experimental Analysis of Behavior* 67(3)**,** 353-366. doi: 10.1901/jeab.1997.67-353.
- Richards, J.B., Zhang, L., Mitchell, S.H., and De Wit, H. (1999). Delay or probability discounting in a model of impulsive behavior: effect of alcohol. *Journal of the experimental analysis of behavior* 71(2)**,** 121-143. doi: 10.1901/jeab.1999.71-121.
- Steinglass, J.E., Figner, B., Berkowitz, S., Simpson, H.B., Weber, E.U., and Walsh, B.T. (2012). Increased capacity to delay reward in anorexia nervosa. *Journal of the International Neuropsychological Society* 18(4), 773-780. doi: 10.1017/S1355617712000446.
- Sugiwaka, H., and Okouchi, H. (2004). Reformative self‐control and discounting of reward value by delay or effort 1. *Japanese Psychological Research* 46(1), 1-9. doi: 10.1111/j.1468-5884.2004.00231.x.
- Wang, Y., Hu, Y., Xu, J., Zhou, H., Lin, X., Du, X., et al. (2017a). Dysfunctional prefrontal function is associated with impulsivity in people with internet gaming disorder during a delay discounting task. *Frontiers in Psychiatry* 8**,** 287. doi: 10.3389/fpsyt.2017.00287.
- Wang, Q., Luo, S., Monterosso, J., Zhang, J., Fang, X., Dong, Q., & Xue,G. (2014). Distributed value representation in the medial prefrontalcortex during intertemporal choices.The Journal of Neuroscience,34(22), 7522-7530. Doi: 10.1523/JNEUROSCI.0351-14.2014

**Supplementary Table 3.**

*Complete Meta-Analytic Results by Addiction Category*

| **Study** | **d** | **95% CI Lower** | **95% CI Upper** | **Z** | ***p*** | **OSR** | **OSR-*p*** |
| --- | --- | --- | --- | --- | --- | --- | --- |
| **CATEGORICAL DESIGNS** |  |  |  |  |  |  |  |
| **Gambling** |  |  |  |  |  |  |  |
| Albein-Urios 2012 | 1.29 | 0.63 | 1.95 | 3.85 | <0.001 | 0.83 | <0.001 |
| Albein-Urios 2014 | 0.46 | -0.04 | 0.97 | 1.82 | 0.069 | 0.87 | <0.001 |
| Brevers 2012 | 1.21 | 0.67 | 1.76 | 4.36 | <0.001 | 0.84 | <0.001 |
| Calluso 2020 | 3.91 | 2.93 | 4.88 | 7.85 | <0.001 | 0.76 | <0.001 |
| Ciccarelli 2016 | 0.42 | 0.04 | 0.81 | 2.18 | 0.029 | 0.87 | <0.001 |
| Contrereas-Rodriguez 2015 | 0.53 | -0.10 | 1.16 | 1.64 | 0.101 | 0.86 | <0.001 |
| Cosenza 2017 | 0.53 | 0.13 | 0.92 | 2.63 | 0.008 | 0.87 | <0.001 |
| DeWilde 2013 | 0.70 | 0.14 | 1.27 | 2.45 | 0.014 | 0.86 | <0.001 |
| Dixon 2003 | 2.19 | 1.40 | 2.97 | 5.47 | <0.001 | 0.80 | <0.001 |
| Freinhofer 2020 | 0.47 | -0.10 | 1.04 | 1.60 | 0.109 | 0.86 | <0.001 |
| Hiraoka 2018 | 1.45 | 0.28 | 2.63 | 2.42 | 0.016 | 0.84 | <0.001 |
| Holt 2003 | 0.68 | 0.03 | 1.33 | 2.04 | 0.042 | 0.86 | <0.001 |
| Holt 2003b | 0.25 | -0.39 | 0.88 | 0.75 | 0.451 | 0.87 | <0.001 |
| Kraplin 2014 | 0.95 | 0.28 | 1.62 | 2.77 | 0.006 | 0.85 | <0.001 |
| Ledgerwood 2009 | 1.11 | 0.60 | 1.61 | 4.29 | <0.001 | 0.84 | <0.001 |
| MacKillop 2006 | 0.69 | 0.14 | 1.23 | 2.47 | 0.013 | 0.86 | <0.001 |
| Madden 2009 | 0.19 | -0.45 | 0.83 | 0.58 | 0.561 | 0.87 | <0.001 |
| Michalczuk 2011 | 0.43 | -0.09 | 0.96 | 1.61 | 0.107 | 0.87 | <0.001 |
| Miedl 2012 | 0.98 | 0.25 | 1.71 | 2.62 | 0.009 | 0.85 | <0.001 |
| Miedl 2015 | 0.66 | -0.07 | 1.40 | 1.77 | 0.077 | 0.86 | <0.001 |
| Mohammadi 2016 | 0.66 | -0.07 | 1.40 | 1.77 | 0.077 | 0.86 | <0.001 |
| Nigro 2016 | 0.61 | 0.27 | 0.95 | 3.51 | <0.001 | 0.86 | <0.001 |
| Secades-Villa 2016 | -0.10 | -0.41 | 0.21 | -0.60 | 0.548 | 0.88 | <0.001 |
| Torres 2013 | 1.50 | 0.83 | 2.17 | 4.40 | 0.000 | 0.83 | <0.001 |
| Weidacker 2020 | 0.39 | -0.39 | 1.16 | 0.97 | 0.331 | 0.86 | <0.001 |
| Wiehler 2015 | 0.35 | -0.27 | 0.98 | 1.11 | 0.268 | 0.87 | <0.001 |
| Williams 2012 | 1.04 | 0.45 | 1.63 | 3.44 | 0.001 | 0.84 | <0.001 |
| Wolfling 2020b | 1.10 | 0.54 | 1.65 | 3.89 | <0.001 | 0.84 | <0.001 |
|  |  |  |  |  |  |  |  |
| **IGD (Categorical)** |  |  |  |  |  |  |  |
| Buono 2017 | 0.83 | 0.33 | 1.34 | 3.24 | 0.001 | 0.82 | <0.001 |
| Buono 2017b | 0.53 | 0.04 | 1.02 | 2.11 | 0.035 | 0.85 | <0.001 |
| Irvine 2013 | 1.70 | 1.06 | 2.34 | 5.20 | 0.000 | 0.82 | <0.001 |
| Raiha 2020 | 3.54 | 2.75 | 4.33 | 8.77 | 0.000 | 0.59 | <0.001 |
| Tian 2018 | 0.41 | -0.02 | 0.85 | 1.86 | 0.062 | 0.86 | <0.001 |
| Tian 2018b | 0.75 | 0.30 | 1.19 | 3.30 | 0.001 | 0.83 | <0.001 |
| Wang 2017a | 0.64 | 0.00 | 1.29 | 1.95 | 0.051 | 0.84 | <0.001 |
| Wang 2017b | 0.43 | -0.20 | 1.05 | 1.35 | 0.179 | 0.86 | <0.001 |
| Wang 2017c | 0.53 | -0.10 | 1.16 | 1.65 | 0.098 | 0.85 | <0.001 |
| Weinstein 2016 | 0.79 | 0.15 | 1.43 | 2.41 | 0.016 | 0.83 | <0.001 |
| Wolfling 2020 | 0.51 | -0.02 | 1.04 | 1.90 | 0.058 | 0.85 | <0.001 |
| Yao 2017 | 1.01 | 0.40 | 1.63 | 3.22 | 0.001 | 0.81 | <0.001 |
| Yan 2021 | 0.41 | 0.04 | 0.78 | 2.17 | 0.030 | 0.87 | <0.001 |

**Supplementary Table 3 (Continued)**

| **Study** | **d** | **95% CI Lower** | **95% CI Upper** | **Z** | ***p*** | **OSR** | **OSR-*p*** |
| --- | --- | --- | --- | --- | --- | --- | --- |
| **Internet / Smartphone (Categorical)** | | |  |  |  |  |  |
| Delaney 2018b | 0.42 | -0.14 | 0.98 | 1.48 | 0.138 | 0.12 | 0.264 |
| Hayashi 2021b | 0.22 | -0.34 | 0.78 | 0.76 | 0.446 | 0.16 | 0.177 |
| Li 2016 | 0.76 | 0.21 | 1.30 | 2.73 | 0.006 | 0.03 | 0.670 |
| Liu 2019 | 0.04 | -0.13 | 0.21 | 0.49 | 0.623 | 0.27 | 0.124 |
| Liu 2019b | -0.07 | -0.25 | 0.11 | -0.75 | 0.453 | 0.28 | 0.054 |
| Saville 2010 | 0.26 | -0.49 | 1.00 | 0.68 | 0.497 | 0.16 | 0.171 |
|  |  |  |  |  |  |  |  |
| **DIMENSIONAL DESIGNS** |  |  |  |  |  |  |  |
| **Study**  (**Clinical-only sample) | **r** | **95% CI Lower** | **95% CI Upper** | **Z** | ***p*** | **OSR** | **OSR-*p*** |
| **Gambling (Dimensional)** |  |  |  |  |  |  |  |
| Alessi 2003** | 0.47 | 0.25 | 0.64 | 3.92 | <0.001 | 0.21 | <0.001 |
| Alessi 2003b** | 0.34 | 0.10 | 0.54 | 2.72 | 0.007 | 0.21 | <0.001 |
| Alessi 2003c** | 0.42 | 0.19 | 0.61 | 3.44 | 0.001 | 0.21 | <0.001 |
| Andrade 2014** | 0.22 | 0.11 | 0.32 | 3.95 | <0.001 | 0.22 | <0.001 |
| Andrade 2014b** | 0.18 | 0.07 | 0.28 | 3.21 | 0.001 | 0.22 | <0.001 |
| Awo 2021 | 0.43 | 0.30 | 0.54 | 6.01 | <0.001 | 0.21 | <0.001 |
| Callan 2011 | 0.24 | 0.03 | 0.43 | 2.19 | 0.029 | 0.22 | <0.001 |
| Canale 2015 | 0.08 | 0.02 | 0.14 | 2.51 | 0.012 | 0.23 | <0.001 |
| Ciccarelli 2016b | 0.21 | 0.02 | 0.38 | 2.18 | 0.029 | 0.22 | <0.001 |
| Ciccarelli 2019 | 0.55 | 0.42 | 0.66 | 6.98 | <0.001 | 0.20 | <0.001 |
| Cosenza 2015 | 0.17 | 0.11 | 0.23 | 5.53 | <0.001 | 0.22 | <0.001 |
| Gray 2014 | 0.34 | 0.20 | 0.46 | 4.64 | <0.001 | 0.21 | <0.001 |
| Jousta 2015 | 0.22 | -0.41 | 0.70 | 0.67 | 0.502 | 0.22 | <0.001 |
| MacKillop 2014 | 0.27 | 0.17 | 0.36 | 5.18 | <0.001 | 0.21 | <0.001 |
| Mishra 2017 | 0.25 | 0.15 | 0.35 | 4.60 | <0.001 | 0.22 | <0.001 |
| Montes 2017 | 0.28 | -0.01 | 0.53 | 1.86 | 0.062 | 0.22 | <0.001 |
| Nigro 2017 | 0.11 | 0.05 | 0.17 | 3.44 | 0.001 | 0.22 | <0.001 |
| Petry 2001** | 0.36 | 0.12 | 0.56 | 2.85 | 0.004 | 0.21 | <0.001 |
| Petry 2012** | 0.02 | -0.11 | 0.15 | 0.30 | 0.765 | 0.23 | <0.001 |
| Schluter 2018** | 0.05 | -0.13 | 0.23 | 0.53 | 0.595 | 0.22 | <0.001 |
| Stea 2011 | 0.21 | 0.08 | 0.33 | 3.12 | 0.002 | 0.22 | <0.001 |
| Tabri 2017 | 0.31 | 0.19 | 0.42 | 4.92 | <0.001 | 0.21 | <0.001 |
| Thomas 2015 | 0.01 | -0.11 | 0.13 | 0.23 | 0.818 | 0.23 | <0.001 |
| Thomas 2015b | 0.00 | -0.11 | 0.12 | 0.07 | 0.948 | 0.23 | <0.001 |
| Yan 2016 | 0.02 | -0.04 | 0.08 | 0.60 | 0.547 | 0.23 | <0.001 |
|  |  |  |  |  |  |  |  |
| **Internet / Smartphone (Dimensional)** | | |  |  |  |  |  |
| Acuff 2021 | 0.08 | 0.03 | 0.13 | 2.94 | 0.003 | 0.16 | <0.001 |
| Acuff 2021b | 0.03 | -0.03 | 0.08 | 1.04 | 0.301 | 0.16 | <0.001 |
| Antons 2019 | 0.07 | 0.01 | 0.12 | 2.52 | 0.012 | 0.16 | <0.001 |
| Delaney 2018 | 0.26 | 0.04 | 0.46 | 2.26 | 0.024 | 0.12 | <0.001 |
| Hayashi 2020 | 0.20 | 0.05 | 0.34 | 2.60 | 0.009 | 0.12 | 0.001 |
| Schulz Van Endert 2020 | 0.21 | 0.02 | 0.39 | 2.11 | 0.035 | 0.12 | <0.001 |
| Schulz Van Endert 2021 | 0.27 | 0.05 | 0.47 | 2.35 | 0.019 | 0.12 | 0.001 |
| Turel 2018 | 0.63 | 0.37 | 0.80 | 4.03 | <0.001 | 0.10 | <0.001 |
| Vargas 2019 | 0.28 | -0.03 | 0.54 | 1.77 | 0.076 | 0.12 | <0.001 |
| Wilmer 2019 | -0.03 | -0.22 | 0.16 | -0.30 | 0.761 | 0.15 | <0.001 |

**Supplementary Table 3 (Continued)**

| **Study** | **r** | **95% CI Lower** | **95% CI Upper** | **Z** | ***p*** | **OSR** | **OSR-*p*** |
| --- | --- | --- | --- | --- | --- | --- | --- |
| **Food Addiction (Dimensional)** | | |  |  |  |  |  |
| Kekic 2019 | 0.16 | 0.07 | 0.25 | 3.33 | 0.001 | 0.17 | 0.057 |
| Minhas 2021a | 0.04 | -0.01 | 0.09 | 1.51 | 0.131 | 0.19 | <0.001 |
| Minhas 2021b | 0.05 | -0.02 | 0.12 | 1.35 | 0.178 | 0.15 | 0.008 |
| Peng-Li 2020 | 0.33 | 0.09 | 0.53 | 2.68 | 0.007 | 0.12 | 0.025 |
| VanderBroek-Stice 2017 | 0.21 | 0.07 | 0.35 | 2.84 | 0.004 | 0.14 | 0.039 |

*Note.* CI = confidence interval; IGD = Internet Gaming Disorder; OSR = aggregate effect size when each study was omitted in one study removed analysis

**Sample was restricted to clinical participants only (e.g., individuals meeting criteria for gambling disorder or a history of gambling problems)

**Supplementary Figure 1**

*Funnel Plots Depicting Imputed Studies for Dimensional Gambling and Internet Smartphone Categories.* Note: Observed studies in the meta-analytic sample indicated by unfilled circles; imputed missing studies (identified using a random-effects model) indicated by filled circles. Aggregate effect size (Fisher’s Z) depicted at bottom of funnel plot, with unfilled diamond depicting the observed effect size and filled diamond depicting the adjusted effect size including imputed studies.

**A) Gambling**


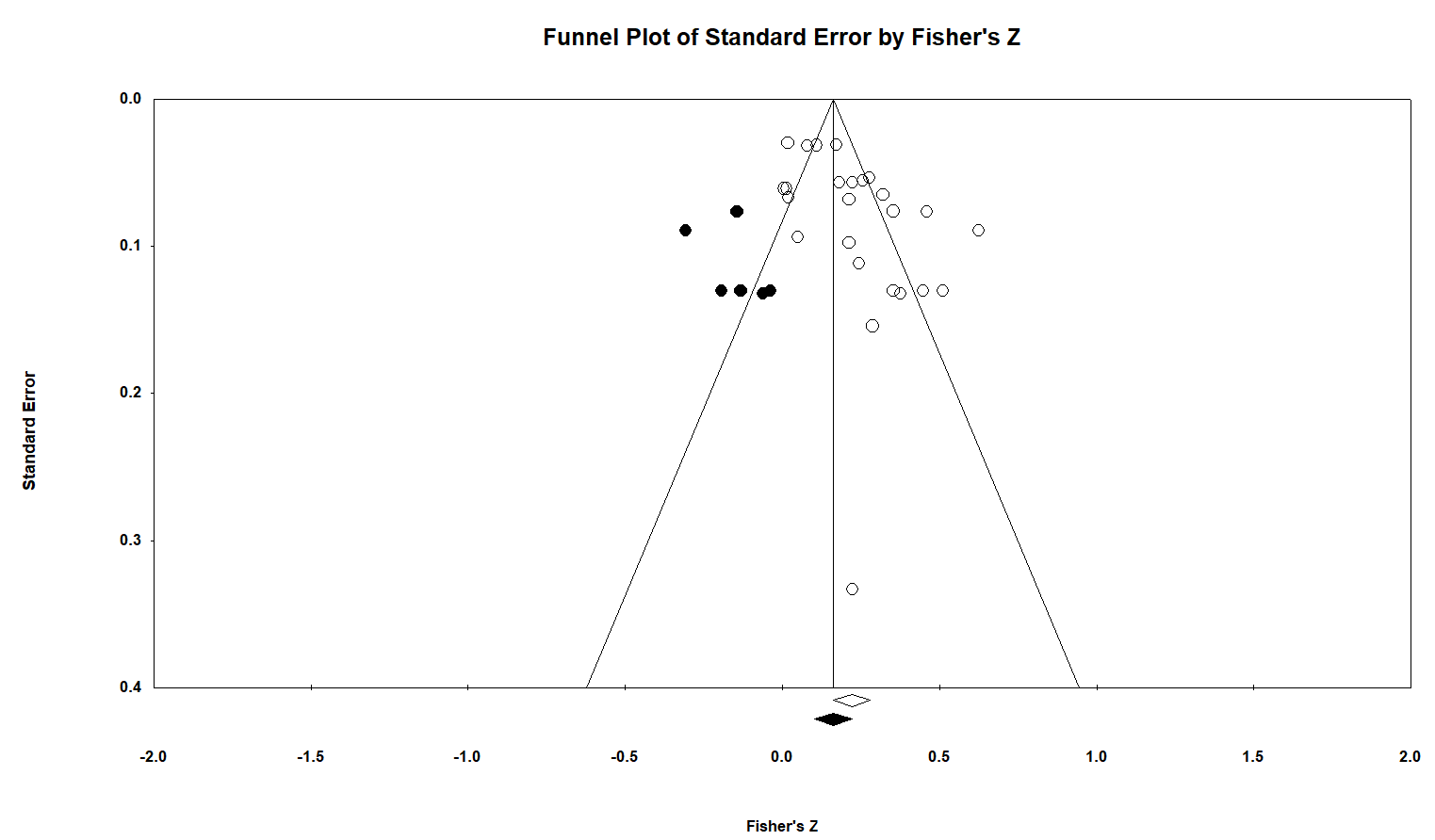


**B) Internet Smartphone**


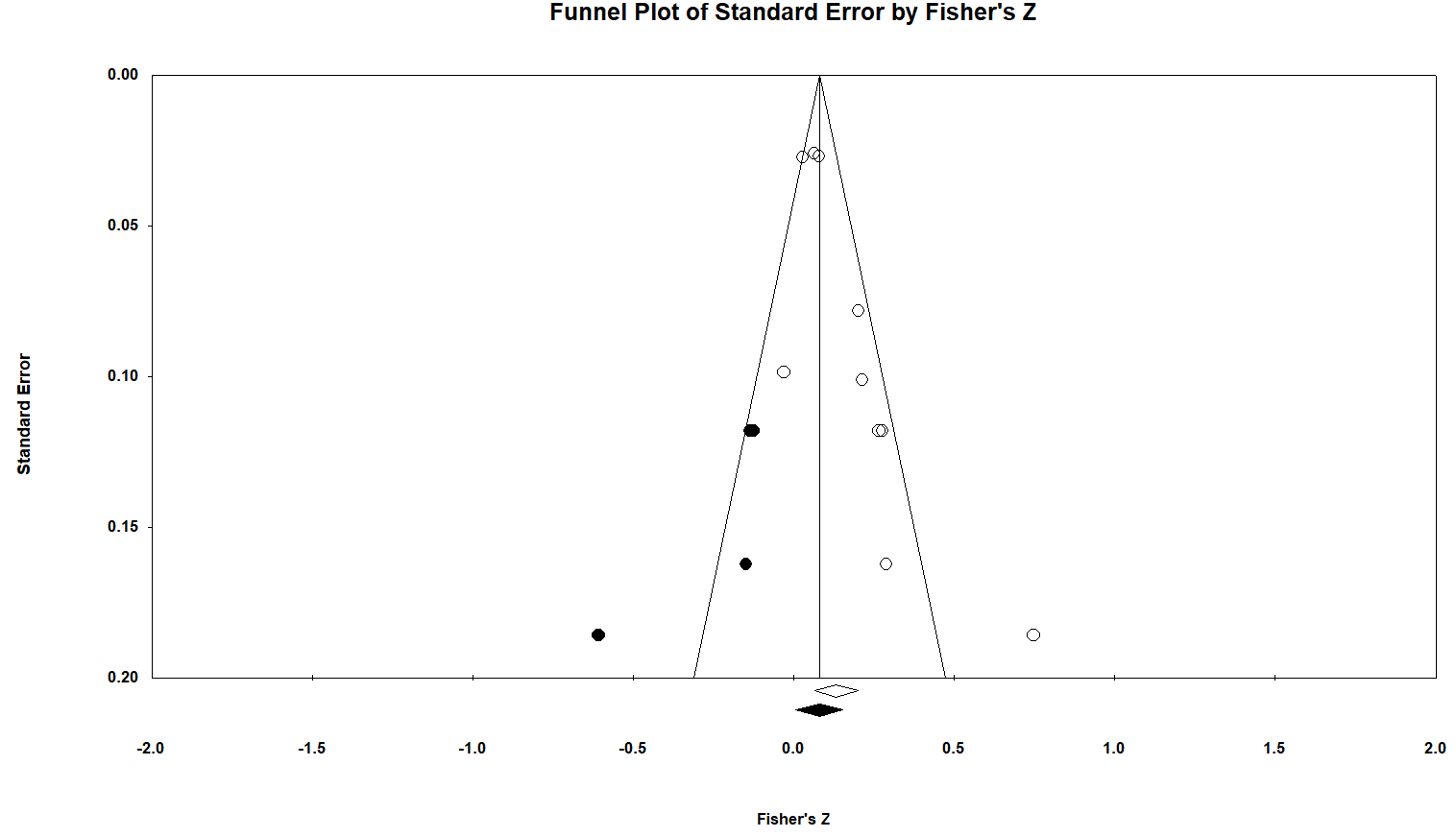

Supplement: Supplementary file 1 [file Table_1.DOCX]
